# Development of a New Index to Distinguish Hepatic Encephalopathy through Automated Quantification of Globus Pallidal Signal Intensity Using MRI

**DOI:** 10.3390/diagnostics12071584

**Published:** 2022-06-29

**Authors:** Yasuyuki Tamai, Motoh Iwasa, Yuichi Yoshida, Jun Nomoto, Takahiro Kato, Hiroe Asuke, Akiko Eguchi, Yoshiyuki Takei, Hayato Nakagawa

**Affiliations:** 1 Department of Gastroenterology and Hepatology, Mie University Graduate School of Medicine, Tsu 514-8507, Japan; tamai304051@clin.medic.mie-u.ac.jp (Y.T.); motoh@med.mie-u.ac.jp (M.I.); ytakei@clin.medic.mie-u.ac.jp (Y.T.); nakagawah@med.mie-u.ac.jp (H.N.); 2 Department of Gastroenterology and Hepatology, Suita Municipal Hospital, Osaka 564-8567, Japan; yoshida0759@mhp.suita.osaka.jp; 3 HAMATO Neurosurgery Clinic, Yokohama 236-0052, Japan; jun.nomoto@gmail.com; 4 Soubudai Neurosurgical Clinic, Sagamihara 252-0324, Japan; takahiro1976@me.com; 5 Medical Affairs Department, ASKA Pharmaceutical Co., Ltd., Tokyo 108-8532, Japan; asuke-h@aska-pharma.co.jp

**Keywords:** globus pallidus, MRI, hepatic encephalopathy, liver cirrhosis, dementia

## Abstract

Hyperintensities within the bilateral globus pallidus on T1-weighted magnetic resonance images were present in some liver cirrhosis patients with hepatic encephalopathy. The symptoms of covert hepatic encephalopathy are similar to those of mild dementia. We aimed to develop a new diagnostic index in which to distinguish hepatic encephalopathy from dementia. The globus pallidus signal hyperintensity was quantified using three-dimensional images. In addition, the new index value distribution was evaluated in a cohort of dementia patients. Signal intensity of globus pallidus significantly increased in liver cirrhosis patients with hepatic encephalopathy compared to those without hepatic encephalopathy (*p* < 0.05), healthy subjects (*p* < 0.05) or dementia patients (*p* < 0.001). Only 12.5% of liver cirrhosis patients without hepatic encephalopathy and 2% of dementia patients exceeded the new index cut-off value of 0.994, which predicts hepatic encephalopathy. One dementia patient in our evaluation had a history of liver cancer treatment and was assumed to have concomitant hepatic encephalopathy. The automatic assessment of signal intensity in globus pallidus is useful for distinguishing liver cirrhosis patients with hepatic encephalopathy from healthy subjects and liver cirrhosis patients without hepatic encephalopathy. Our image analyses exclude possible cases of hepatic encephalopathy from patients with neurocognitive impairment, including dementia.

## 1. Introduction

Dementia is a clinical syndrome characterized by a progressive deterioration in cognitive ability along with a diminished capacity for independent living and functioning [1]. When physicians suspect dementia, they refer the patient to a neurologist who performs a diagnostic examination that includes neuroimaging. Magnetic resonance imaging (MRI) is particularly necessary at least once during the diagnostic work-up of patients with suspected or diagnosed dementia [2] due to its utility in identifying or excluding potentially treatable causes of dementia [3,4]. Indeed, approximately 10–30% of patients diagnosed with dementia are treatable (e.g., normal pressure hydrocephalus, vitamin B12 deficiency [4,5]); however, very few patients with hepatic encephalopathy (HE) can be included in this category. A clinical diagnosis of HE is reached after all other possible causes of brain dysfunction have been excluded, and underlying liver cirrhosis (LC) must also be confirmed [6]. However, in some patients with LC, hepatic dysfunction is very mild; even in the presence of cognitive dysfunction, these cognitive disturbances are not easily distinguished from forms of dementia such as Alzheimer’s disease.

Characteristic MRI observations associated with chronic HE include cerebral atrophy and bilateral symmetric hyperintensities of the globus pallidus (GP) on T1-weighted images without corresponding signal intensities in T2-weighted images [7]. This signal hyperintensity is thought to be due to the deposition of manganese associated with the portosystemic shunt and decreased hepatobiliary clearance of metabolites from the intestines [8]. An accumulation of manganese will contribute to the dysfunction of neurons and astrocytes leading to HE [9]. Moreover, Pomier-Layrargue et al. reported that pallidal manganese concentration is markedly elevated in LC patients who died in a state of hepatic coma [10]. Therefore, the quantification of GP signal intensity on T1-weighted MRI observations is incredibly valuable for diagnosing patients with HE.

The signal intensity of GP is detected by subjectively selecting regions of interest chosen by the operator [7,8,11,12,13]; therefore, there is a possibility of discrepancies between operators. Therefore, computer automation is desirable insofar as it allows for objectivity in judgment. Furthermore, HE is easily overlooked by clinicians due to a very low prevalence and some LC patients presenting with normal alanine aminotransferase (ALT) levels. As a consequence, HE can be present in patients clinically diagnosed with dementia. A tool that can automatically detect the signal hyperintensity of GP on T1-weighted images can assist physicians in making a more accurate imaging diagnosis, consequently reducing the overall physician workload. 

In this study, we focused on the high signal of GP in T1-weighted MRI results from LC patients with and without HE, and measured the signal intensity of GP from the three-dimensional (3D) T1 images using a brain MRI image analysis program, which was developed for this study. We further quantified the signal hyperintensity and established a new diagnostic index to distinguish HE from other brain-related cognitive diseases. In addition, we utilized existing 3D T1 images obtained from patients previously diagnosed with Alzheimer’s disease or vascular dementia and analyzed the distribution of data based on our newly created index parameters.

## 2. Methods

### 2.1. Study Population

This is a retrospective, observational, multicenter study. Prior to the initiation of the study, the study protocol was reviewed and approved by the clinical research ethics review committee of Mie University hospital (Approval No. H2019-208, approved date: 5 December 2019), the Suita municipal hospital clinical research ethics committee (Approval No. 2019-26, approved date: 21 February 2020), and the Asai dermatology institutional review board (Approval No. 20200217-2, approved date: 17 February 2020). Since this was a retrospective medical record survey, it was exempt from written or oral consent; however, we released information on this research so that patients could opt out of having their data used. Administrative permissions and licenses were acquired by our team to access the data used in our research. All methods were performed in accordance with the relevant guidelines and regulations.

A total of 26 LC patients, who were hospitalized from December 2018 to January 2020 in the Department of Gastroenterology and Hepatology, Mie University Hospital, were included in this study. Patients positive for hepatitis B surface antigen were diagnosed with HBV infection, whereas those positive for hepatitis C virus (HCV) RNA were diagnosed with HCV infection. Alcohol-associated liver disease was defined as alcohol consumption >60 g/day. Nonalcoholic steatohepatitis was diagnosed based on pathological findings and/or fatty liver without any other evident causes of chronic liver disease (viral, autoimmune, genetic, etc.). The diagnosis of LC was based on the aspartate aminotransferase (AST)-to-platelet ratio index (>2) [14], diagnostic imaging such as computed tomography, and endoscopic identification of esophageal varices, or liver biopsies. Ten patients exhibited overt HE, and the remaining 16 had no previous history of overt HE. Overt HE was diagnosed per the established West Haven criteria [15]. The exclusion criteria for our study were cardiac and/or respiratory failure, renal failure with serum creatinine >2 mg/dL, recent excessive alcohol intake, clinical or biochemical signs of infection 1 month prior to inclusion, and a neurological or psychiatric disorders. A blood test was conducted to measure albumin, total bilirubin, AST, ALT, creatinine, prothrombin time (PT), and ammonia. Healthy subjects (*n* = 11) without chronic diseases or regular medication use, excessive alcohol consumption, and those free of acute illnesses within the last month served as control subjects. Firstly, we established the new cut-off value of GP intensity predicting HE. In LC patients without HE, we also investigated the incidence of patients who exceeded the cut-off value obtained from HE.

Next, using this new cut-off value of GP intensity, we further examined the brain MRI from 250 patients with dementia, mainly Alzheimer’s disease, or vascular dementia clinically diagnosed between January 2010 and June 2019. The two neurology outpatient clinics specializing in dementia collaborated in this study. Cases were referred to these clinics for assessment, diagnosis and management by their primary care physician. All diagnoses of dementia were made according to the Diagnostic and Statistical Manual of Mental Disorders (DSM-IV) criteria. Patients with LC, evidence of other neurodegenerative disorders other than Alzheimer’s disease, mild cognitive impairment, cognitive impairment resulting from cerebral trauma, hypoxic cerebral damage, cerebral neoplasia, metabolic disease, or intellectual disability were excluded. In this dementia cohort, we investigated the incidence of patients who exceeded the cut-off value obtained from HE. 

### 2.2. MRI Protocols

Brain MRI was performed on a 3-T MR scanner with a 32-channel phased-array head coil or a dStream HeadNeckSpine coil (Ingenia, Philips Health Care, Best, The Netherlands) including sagittal 3D T1-weighted imaging. The 3D T1-weighted image parameters included field of view (FOV), 260 × 236 mm; matrix, 384 × 384; section thickness, 0.9 mm; TR (ms)/TE (ms) ratio, 8.2 (shortest); TE (shortest), 4.6 ms; flip angle, 10°; and an acquisition time of 4 min 42 s. Other MR sequence parameters were as follows: DWI: TR/TE, 5600/87 ms; FOV, 220 × 220 mm; slice thickness, 3 mm; b value, 1000 s/mm^2^; and scan time, 71 s; 2D T2-weighted images: TR/TE, 8000/90 ms; TSE factor, 15; FOV, 220 × 197 mm; slice thickness/gap = 3/0.5 mm; and scan time, 2 min 48 s. Equivalent MRI protocols are also selected for patients with dementia.

### 2.3. Image Analyses

**Figure 1:**
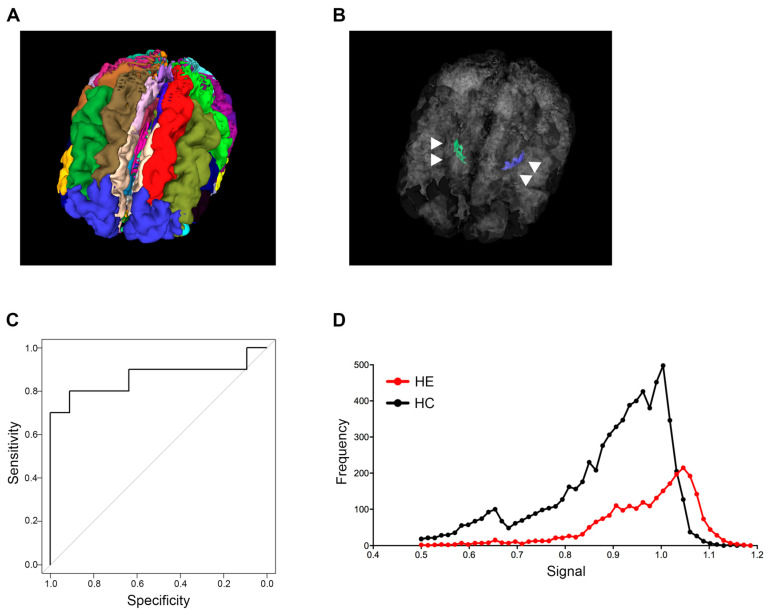
Globus pallidus intensity predicting hepatic encephalopathy. (**A**) Thirty brain segments using the MRI-TAISEKI software and (**B**) extraction of GP (arrowhead) volume data from 133 segmented regions in a 76-year-old female patient with Alzheimer’s disease. (**C**) ROC curves for predicting HE based on signal intensity compared to healthy subjects. The 60th percentile value of global intensity normalization had an AUC of 0.864. (**D**) Representative signal intensity histograms of a patient with HE (68-year-old male) and a healthy subject (74-year-old male). GP, globus pallidus; ROC, receiver operator characteristic; HE, hepatic encephalopathy; AUC, area under the curve.

The analysis of sagittal 3D T1-weighted images in this study was performed by modifying the algorithm in the MRI-TAISEKI software developed by Nippontect Systems Co., Ltd. (Tokyo, Japan). This segmentation procedure incorporates a joint-label fusion method (JLF) [16] and corrective learning (SegAdapter) [17], with 30 manually traced atlases (Figure 1A). Each atlas consisted of 131 manually traced labels within the voxel of interest (VOIs) and a corresponding T1-weighted image. 

Briefly, the segmentation procedure involves the following algorithms: (i) The target T1-weighted images were divided into 30 large regions by non-linear warping from the large-region atlas in the Montreal Neurological Institute (MNI) space, prepared in advance, and the target T1-weighted image; (ii) The top 5 atlases at each large region with the highest Pearson correlation coefficients between the 30 atlases and the target image were selected; (iii) A diffeomorphic anatomical registration through exponentiated lie algebra (DARTEL) template was created from the 5 selected atlases and the target image at each large-region. Subsequently, the five atlases, labels and T1-weighted images were non-linear warped to the target image via the large-region DARTEL templates; (iv) Using JLF and SegAdapter, the target image was segmented into 133 labels by fusing selected atlases at each large region; (v) The signal intensity of T1-weighted images after global (or local) intensity normalization performed using Computational Anatomy Toolbox 12 (CAT12, Structural Brain Imaging Group, University of Jena, Jena, Germany) were extracted within each label (http://www.neuro.uni-jena.de/cat/ (accessed on October 2020)). In this study, we extracted the volume data of GP from 133 segmented regions. Two types of signal intensity normalization methods provided by CAT12 (global intensity normalization; described as “G” and local intensity normalization; described as “L”) were applied to GP measurements. We launched CAT12 using the expert mode and ran the “segmentation” pipeline. We set output parameters for ‘Bias, noise and global intensity corrected T1 image-Native space’ and ‘Bias, noise and local intensity corrected T1 image-Native space’ to “yes” in order to obtain both the global intensity and local intensity corrected images in the native space.

### 2.4. Statistical Analysis

Continuous variables are presented as median and interquartile range, and categorical variables are shown as number of patients [18]. The continuous data were compared using a Student’s *t-*test. The categorical data were compared using a chi-squared test. The median, 60th percentile, 70th percentile, 80th percentile value, and Hodges–Lehmann estimator were calculated for the signal distribution in the GP measurements. Receiver operator characteristic (ROC) curves and the corresponding area under the curve (AUC) were used to obtain cut-offs for the outcomes. The Youden index was applied to calculate the optimal cut-off point. One-way analysis of variance test and Tukey’s honest significant difference test were performed to determine the difference between the four groups. All statistical analyses were performed using SPSS 21.0 software (IBM, Armonk, NY, USA). All tests were two-tailed, and *p* < 0.05 was considered significant.

## 3. Results

### 3.1. Subjects

Baseline clinical and laboratory characteristics of our patient cohort are shown in Table 1. Age and distribution of gender had no significant differences among the three groups. In LC patients with HE, total bilirubin, PT and ammonia levels were 2.2 ± 2 mg/dL, 59.7 ± 24.5% and 112 ± 46 μg/dL, respectively, indicating that the majority of patients with HE presented with more severe liver damage when compared to LC patients without HE.

### 3.2. Establishment of the Cut-Off Value for GP Intensity Predicting HE

Signal intensity variability of the GP in T1-weighted images was successfully quantified for all patients and healthy subjects. Thirty manually traced atlases are shown in Figure 1A, and the anatomical location of the GP structure from a representative case in a 3D rendering is shown in Figure 1B. The AUC and cut-off values that distinguish between healthy subjects and LC patients with HE were calculated for each percentile grouping (Table 2). Image type “global intensity normalization (G)” generally had higher AUC values when compared to the type “local intensity normalization (L)”, and the 60th percentile value of type “G” had the highest AUC value of 0.864. Guided by the above results, we established a cut-off value of 0.994 for the 60th percentile based on type “G” images and adopted this mark as our new index for patient screening (Table 2 and Figure 1C). 

### 3.3. Signal Intensity Histograms in GP from an HE Patient and a Healthy Subject

Representative GP signal intensity histograms from an LC patient with HE and a healthy subject are shown in Figure 1D. The LC patient with HE histogram had a peak in the high intensity bins for GP, while the healthy subject histogram displayed broad intensity bins (Figure 1D).

**Figure 2:**
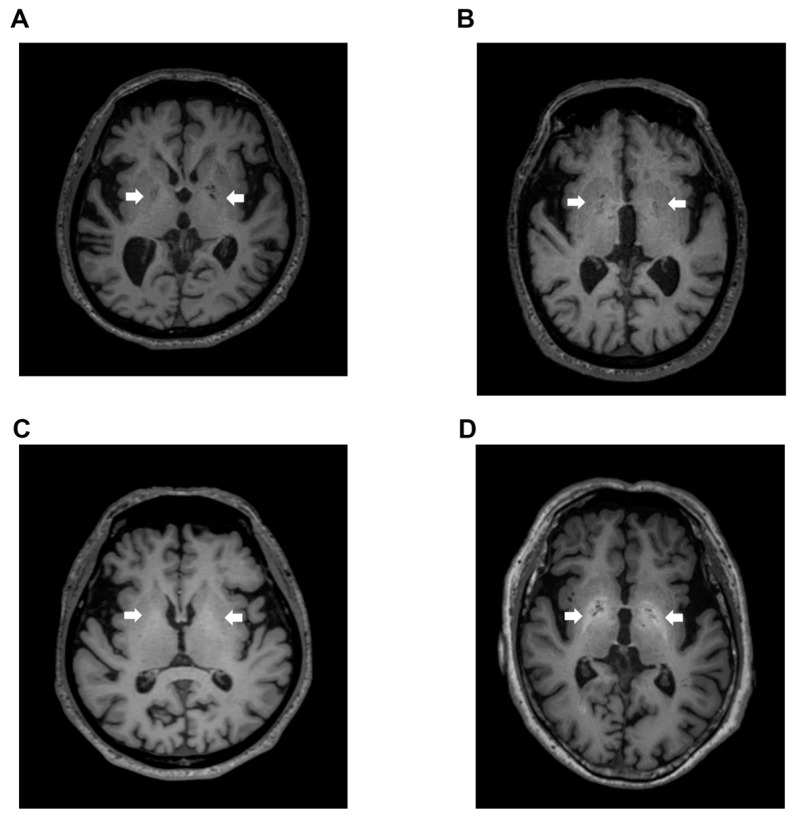
T1-weighted images. (**A**) Transverse T1-weighted imaging from a 74-year-old healthy subject. Signal intensity value of GP (arrow) was 0.952, which is below the cut-off value of 0.994; (**B**) Transverse T1-weighted imaging from a 79-year-old LC patient. Signal intensity value of GP (arrow) was 0.992, also below the cut-off value of 0.994; (**C**) Transverse T1-weighted imaging from a 63-year-old LC patient. Signal intensity value of GP (arrow) was 1.006, which exceeded the cut-off value of 0.994; (**D**) Transverse T1-weighted imaging from a 68-year-old HE patient. Signal intensity value of GP (arrow) was 1.024, also exceeding the cut-off value of 0.994. GP, globus pallidus; LC, liver cirrhosis; HE, hepatic encephalopathy.

**Figure 3:**
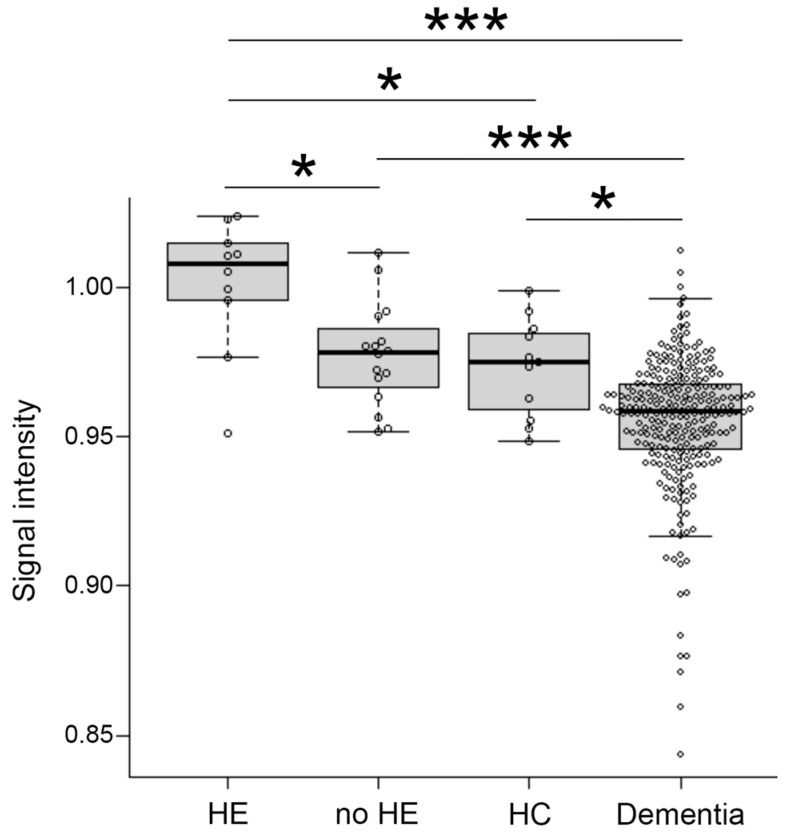
Distribution of signal intensity. The distribution of signal intensity in HE, no-HE, HC and dementia patients. HE, hepatic encephalopathy; HC, healthy control. *: *p* < 0.05, ***: *p* < 0.001.

Signal intensity value of GP (arrow) in transverse T1-weighted imaging was mildly increased (Figure 2B,C) or clearly increased (Figure 2D) on a case-by-case basis, when compared to healthy subject (Figure 2A). Indeed, the signal intensity of GP in LC patients with HE was dramatically increased compared to the values seen in LC patients without HE (*p* < 0.05), healthy subjects (*p* < 0.05), and dementia patients (*p* < 0.001). Contrarily, the signal intensity of GP observed in dementia patients was markedly decreased when compared to those seen in LC patients with HE (*p* < 0.001), LC patients without HE (*p* < 0.001), and healthy subjects (*p* < 0.05) (Figure 3).

### 3.4. Distribution of the New Index in LC without HE and Dementia Patients

Using this new index, we examined the signal value distribution of brain MRI images from 16 LC patients without HE. Only two patients (12.5%) without HE exceeded our established cut-off value of 0.994, which was distinctly different from what was observed in LC patients with HE.

**Figure 4:**
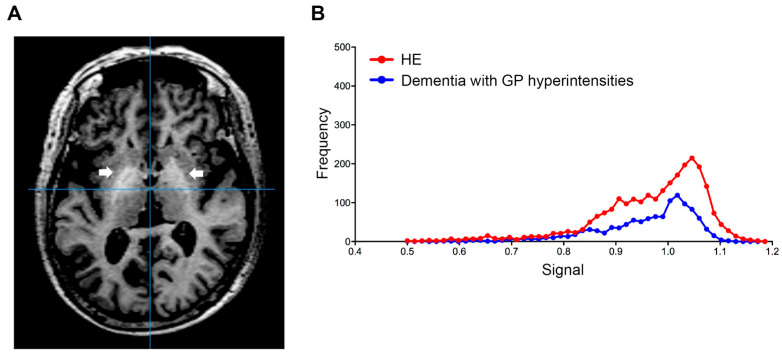
Globus pallidus (arrow) in T1-weighted image. (**A**) Transverse T1-weighted imaging and (**B**) signal intensity histograms of a 76-year-old female patient with Alzheimer’s disease and a history of liver resection due to hepatocellular carcinoma and an HE patient (68-year-old male). Biochemical data from this dementia patient revealed mild thrombocytopenia (11.3 × 10^4^/MCL), mild elevation of transaminases (AST 45 IU/L and ALT 36 IU/L) and diabetes (hemoglobin A1c 7.1%). HE, hepatic encephalopathy; AST, aspartate aminotransferase; ALT, alanine aminotransferase.

Using this index, we also examined the signal value distribution of the brain MRI images from 250 patients with Alzheimer’s disease, or vascular dementia, diagnosed at neurology clinics specializing in dementia. The mean age of the patients was 83.2  ±  6.1 years and 62.4% of the patients were female. Five dementia patients (2%) exceeded our established cut-off value. Therefore, we observed that our automatic assessment of signal intensity in GP is useful to distinguish LC patients with HE from healthy subjects and those without HE. Conversely, signal intensities of GP within the dementia group were markedly decreased; this finding may reflect ischemic changes in the basal ganglia, particularly in patients with vascular dementia (Figure 3). Through a follow-up medical history assessment, it was discovered that one out of five patients who exceeded the cut-off index had undergone liver resection due to hepatocellular carcinoma, and patient biochemical data revealed mild thrombocytopenia (11.3 × 10^4^/MCL), mild elevation of transaminases (AST 45 IU/L and ALT 36 IU/L) and diabetes (hemoglobin A1c 7.1%). This patient displayed severe cerebral atrophy and bilateral symmetric hyperintensities of the GP in T1-weighted images (Figure 4A). The GP intensity histogram of this patient was similar to that of an LC patient with HE (Figure 4B), suggesting the possibility of an unknown hepatic factor concomitant with Alzheimer’s disease. We were unable to confirm obvious signs of LC nor acquire detailed residual data for these four patients.

## 4. Discussion

Symmetric hyperintensities of bilateral GP in T1-weighted images were present in some LC patients with HE [19]. This GP hyperintensity is specific for manganese deposition in LC patients, except for those who received parenteral nutrition or serial administration of gadobutrol [20,21]. This is the first study investigating automated signal intensity quantification of 3D GP structures in patients with HE or dementia. In the present study, the difference in GP signal intensity not only proved useful as a means to distinguish LC patients with HE from healthy subjects (specificity of 0.909 and sensitivity 0.800), but also resulted in the establishment of a new index threshold for LC patients with HE (0.994). Moreover, we confirmed that the majority of LC patients without HE (87.5%) do not exceed the cut-off index, suggesting that GP hyperintensity is related to more severe liver damage in conjunction with HE. 

Diagnostic imaging tools can automatically carry out a quantitative assessment of a large number of patients and achieve greater accuracy in diagnosis with higher efficiency [22]. Our automated image analysis tool enables operators to analyze a large number of cases, as evidenced by the 250 cases analyzed in this study. The novelty of our study is that it shows the utility of our image analyses and its ability to uncover a possible case of HE from patients incorrectly diagnosed with another type of neurocognitive impairment, such as dementia.

Chronic liver disease is frequently associated with overt, or covert HE characterized by a spectrum of neurocognitive dysfunction [23]. Previous reports using magnetic resonance spectroscopy or positron emission tomography demonstrated greater metabolite changes in the basal ganglia in LC patients with HE [24]. These findings may partially explain the clinical characteristics of early stage HE, such as attention deficits, visuo-spatial disorientation and decreased motor speed and accuracy [25]. These symptoms of HE are similar to those of dementia [6]. In patients presenting with clinical characteristics of Alzheimer’s disease, routine imaging of the brain is unlikely to detect any treatable dementia-related factors. In this study, one patient had a history of liver cancer treatment, and the possibility of concomitant hepatic effects, especially HE, was considered.

Despite our important findings, there are a few limitations related to the present study. First, this study was retrospective and the number of healthy subjects and LC patients was low compared to dementia patients. An evaluation of the etiological differences among LC patients is also an important consideration. Furthermore, we did not measure blood ammonia levels in patients with dementia. In this study, four out of five patients who exceeded the cut-off value did not confirm obvious signs of liver disease. Automated assessments of GP hyperintensity in patients with dementia may not be specific for manganese and may reflect the influence of other factors [20,21]. In order to improve our findings, we must accumulate cases and improve accuracy; therefore, it will be important to conduct a large multicenter study in the future.

## 5. Conclusions

The automatic assessment of signal intensity in GP is useful to distinguish LC patients with HE from healthy subjects and LC patients without HE. Our image analyses will exclude possible cases of HE from patients with neurocognitive impairment, whereas it can also distinguish cases of dementia from those with HE. This newly established tool provides the potential to establish the wider clinical use of assessments of GP signal intensity in the management of patients with neurocognitive impairment. 

## Figures and Tables

**Table 1:** Subject demographics.

	Healthy Control (*n* = 11)	Liver Cirrhosis without HE (*n* = 16)	Liver Cirrhosis with HE (*n* = 10)	*p*-Value
Age, years	68 (48–79)	70.5 (63.3–72.8)	65 (55.3–69.5)	0.269
Gender, male/female	8/3	14/2	8/2	0.626
Etiology, HBV/HCV/HBV + HCV/NASH/alcohol/others		2/4/0/9/1/0	1/1/1/2/3/2	0.084
Ammonia, μg/dL	N/A	40 (28–64)	102.5 (76–161.5)	<0.001
Albumin, g/dL	N/A	3.65 (3.3–4.0)	3.2 (2.7–3.7)	0.065
Total bilirubin, mg/dL	N/A	0.8 (0.63–1.18)	1.5 (0.9–2.68)	0.070
AST, IU/L	N/A	44.5 (32.3–67.8)	48.5 (29.8–65.3)	0.400
ALT, IU/L	N/A	27.5 (20.3–70.3)	25 (23–41.3)	0.230
Creatinine, mg/dL	N/A	0.74 (0.68–0.85)	0.69 (0.57–1.05)	0.855
Prothrombin time, %	N/A	97 (83.8–102.9)	56.2 (38.5–79.1)	<0.001

All data represent median (interquartile range) unless otherwise indicated. HE, hepatic encephalopathy; HBV, hepatitis B virus; HCV, hepatitis C virus; NASH, non-alcoholic steatohepatitis; AST, aspartate transaminase; ALT, alanine transaminase; N/A, not available. *p*-values are calculated using ANOVA, chi-square test or unpaired t-test appropriately.

**Table 2:** The AUC and cut-off value that can distinguish between control and patients with HE. Global intensity normalization (G).

	AUC	Cut-Off Value	Specificity	Sensitivity
Median	0.818	0.985	0.909	0.700
60th percentile	0.864	0.994	0.909	0.800
70th percentile	0.855	1.009	1.000	0.700
80th percentile	0.832	1.017	1.000	0.700
Hodges–Lehmann estimator	0.745	0.974	1.000	0.600
Median	0.818	0.970	1.000	0.700
60th percentile	0.845	0.988	0.909	0.800
70th percentile	0.845	1.004	1.000	0.700
80th percentile	0.827	1.007	1.000	0.700
Hodges–Lehmann estimator	0.755	0.937	1.000	0.600

Local intensity normalization (L). AUC, area under the curve; HE, hepatic encephalopathy.
